# Methane emissions from trees planted on a closed landfill site

**DOI:** 10.1177/0734242X221086955

**Published:** 2022-04-05

**Authors:** Alice Fraser-McDonald, Carl Boardman, Toni Gladding, Stephen Burnley, Vincent Gauci

**Affiliations:** 1 School of Engineering and Innovation, The Open University, Walton Hall, Milton Keynes, UK; 2 Birmingham Institute of Forest Research (BIFoR), School of Geography, Earth and Environmental Sciences, University of Birmingham, Edgbaston, UK

**Keywords:** Carbon cycle, landfill, tree stem CH_4_, spatial variability, temporal variability, GHG emissions

## Abstract

Trees have morphological adaptations that allow methane (CH_4_) generated below ground to bypass oxidation in aerobic surface soils. This natural phenomenon however has not been measured in a landfill context where planted trees may alter the composition and magnitude of CH_4_ fluxes from the surface. To address this research gap, we measured tree stem and soil greenhouse gas (GHG) emissions (CH_4_ and CO_2_) from a closed UK landfill and comparable natural site, using an off-axis integrated cavity output spectroscopy analyser and flux chambers. Analyses showed average CH_4_ stem fluxes from the landfill and non-landfill sites were 31.8 ± 24.4 µg m^–2^ h^–1^ and –0.3 ± 0.2 µg m^–2^ h^–1^, respectively. The landfill site showed seasonal patterns in CH_4_ and CO_2_ stem emissions, but no significant patterns were observed in CH_4_ and CO_2_ fluxes at different stem heights or between tree species. Tree stem emissions accounted for 39% of the total CH_4_ surface flux (7% of the CO_2_); a previously unknown contribution that should be included in future carbon assessments.

## Graphical abstract

**Graphical Abstract:**
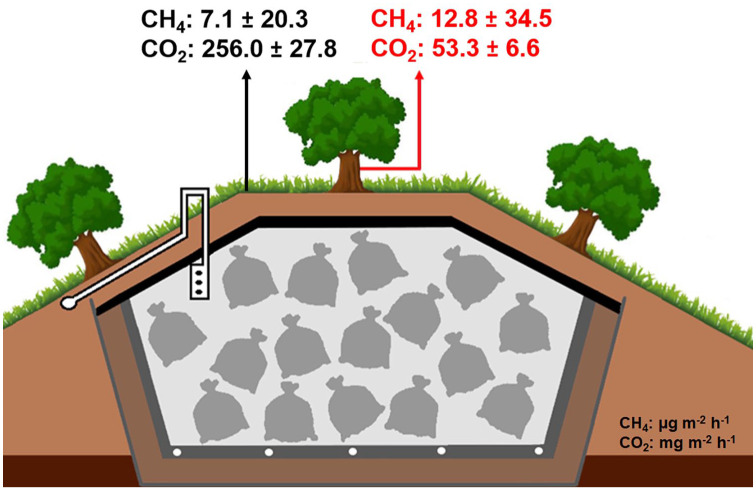

## Introduction

Methane (CH_4_) has directly contributed to 20% of the additional radiative forcing in the lower atmosphere since 1750 and is the greenhouse gas (GHG) with the second-highest radiative forcing after CO_2_ (Ciais et al., 2013; Myhre et al., 2013). Identifying and measuring the global sources and sinks of CH_4_ is important for establishing effective climate change mitigation strategies (Kirschke et al., 2013; Nisbet et al., 2020). Forests play an important role in the global carbon cycle, sequestering globally *ca*.8 Gt CO_2_e yr^–1^ (Harris et al., 2021); however, the significance of trees in facilitating the transportation of subsurface GHGs to the atmosphere has only recently been discovered (Gauci et al., 2010; Pangala et al., 2013).

Trees growing in wetland and upland environments provide a pathway for CH_4_ emissions from underground sources to the atmosphere (Gauci et al., 2010; Maier et al., 2018; Pangala et al., 2013; Terazawa et al., 2007). Morphological adaptations that facilitate the transport of oxygen (O_2_) to roots (e.g. hypertrophied lenticels and enlarged aerenchyma tissue) also aid in the release of CH_4_ from anaerobic soils (Pangala et al., 2017). This pathway allows CH_4_ to bypass oxidation and travel by diffusion or within the transpiration stream of trees until it is emitted from stem surfaces (Pangala et al., 2013). Tree-mediated CH_4_ emissions account for 62%–87% of the total ecosystem CH_4_ flux in a tropical forested ecosystem, and up to 27% of the ecosystem CH_4_ flux in a temperate biome (Pangala et al., 2013, 2015).

The ability of trees to act as conduits for GHGs on landfill sites has not been investigated. CH_4_ is produced in landfills that accepted biodegradable waste through the decomposition of organic matter under anaerobic conditions (Jardine et al., 2006). Landfills and wastewater handling are the third-largest global source of anthropogenic CH_4_, estimated to have released 65 Tg CH_4_ yr^–1^ between 2008 and 2017 (Abushammala et al., 2014; Saunois et al., 2020). Closed sanitary landfill sites have an engineered cap consisting of an impermeable mineral layer, a drainage layer and cover soil; this prevents water infiltration into the waste and landfill gas emissions (Landfill Guidance Group (LGG), 2018). Cover soils have a relatively high concentration of O_2_ that allows methanotrophic bacteria to develop (Boeckx et al., 1996). The quantity of CH_4_ that is oxidised by aerobic bacteria in cover soils ranges from negligible to over 100% (which can result in atmospheric CH_4_ consumption by landfill surfaces) (Abushammala et al., 2014; Boeckx et al., 1996; Bogner et al., 1995, 1997). Methanotrophic oxidation rates in landfill cover soils are related to site management practices; for example, the thickness and composition of cover soil, and seasonal variability in cover soil moisture and temperature (Bogner et al., 1997; Spokas et al., 2011). Planting trees is commonly used as a management strategy to improve the visual appeal of closed landfill sites, increase carbon sequestration and minimise water percolation into waste (Environmental Protection Agency (EPA), 2003; Shah et al., 2017; Venkatraman and Ashwath, 2009). Currently in England, around *ca*.60% of closed landfill sites that accepted household or commercial waste have areas of trees planted on them (Fraser-McDonald, unpublished satellite investigation work).

If tree roots channel CH_4_ from belowground in landfill, the proportion of CH_4_ being oxidised could be reduced, resulting in greater CH_4_ emissions. Trees planted on landfill sites can grow roots that interact with the soil–cap interface (Forest Research, 2008; Hutchings et al., 2001). The interaction and the presence of tree roots in landfill soils may provide a conduit for CH_4_ to bypass oxidation and increase CH_4_ surface emissions. In this study, we investigated this hypothesis by measuring both tree stem and soil surface CH_4_ and CO_2_ fluxes on a landfill and a natural comparison site. The field campaign was designed to investigate spatial and temporal patterns and quantify the contribution of trees to landfill surface fluxes.

## Methods

### Study sites

Our investigation was conducted at two sites in England, UK; a closed landfill site and a non-landfill area in close proximity. The landfill site accepted waste between 1964 and 1998 before being capped and covered with restoration soil. The area was capped with *ca*.1 m minimum of clay with a permeability of 10^–7^ m s^–1^, overlain by *ca*.2 m of topsoil. Leachate and gas control systems are in place, and CH_4_ extracted from the waste is used for energy production. Tree species including *Betula pendula, Fraxinus excelsior* and *Prunus avium* were planted in a 1.22 ha area in 2004. The non-landfill comparison site was a secondary woodland planted in 2003 (7 ha in size) which included the same tree species as the landfill site. *Fraxinus excelsior* was the dominant species but some mature *Betula pendula* and *Prunus avium* trees were also present on both sites; the number of each species measured in our study reflected the composition at each site. Both areas have the same loamy clayey soils with underlying sedimentary bedrock and mudstone, mean annual temperatures (14.1°C) and annual rainfall (565.5 mm) (Lewis et al., 2019).

### Site and tree characteristic measurements

For each tree flux measured the GPS location, tree species and diameter at breast height (DBH) was recorded. Air temperature and air pressure at each location were measured with a Comet C4141 Thermo-hygro barometer. Tree stem surface temperature was recorded using an infrared thermometer (RS Pro RS1327k). At each soil and tree location, soil temperature (Thermapen soil temperature probe) and soil moisture (Delta-T Devices HH2 moisture metre with ThetaProbe type ML2x) were measured at 10 and 6 cm depth, respectively. Soil cores were taken to determine bulk density and pH (Thermo Scientific Orion Versa Star Advanced Electrochemistry metre with Orion 8157 BNUMD ROSS Ultra pH ATC Triode).

### Gas flux measurements

Tree and soil measurements were made at the landfill site between August 2019 and February 2020. Fifteen trees and five soil locations were sampled once a month, with measurements taken at 30, 90 and 150 cm stem heights in August 2019, November 2019 and February 2020 and samples at 90 cm only taken in September 2019, October 2019, December 2019 and January 2020. Gas concentration measurements at the landfill site were taken from 10 *Fraxinus excelsior*, 1 *Prunus avium* and 4 *Betula pendula* trees. An additional 25 trees (stem measurement heights 30 cm, 90 cm and 150 cm) and 19 soil locations were sampled in February 2020 during an intensive fieldwork period. The non-landfill comparison site was visited in February and August 2020 and measurements were taken from 15 trees (30, 90 and 150 cm stem heights) and five soil locations. Gas concentrations at the non-landfill site were measured from 7 *Fraxinus excelsior*, 4 *Prunus avium* and 4 *Betula pendula* trees. The sampling strategy included a suitable number of tree and soil locations to allow appropriate statistical analysis of trace-level GHG exchange and to examine spatial variability across the sites. Flux data for between-site comparisons were taken in different years due to site access restrictions. Kruskal–Wallis tests showed that there was no significant difference in the maximum daily rainfall (mm) and maximum daily temperature (°C) in August between the years 2019 and 2020 or between these years and data from the previous 10-year period (2010–2020), demonstrating that environmental conditions were not atypical.

**Figure 1.**
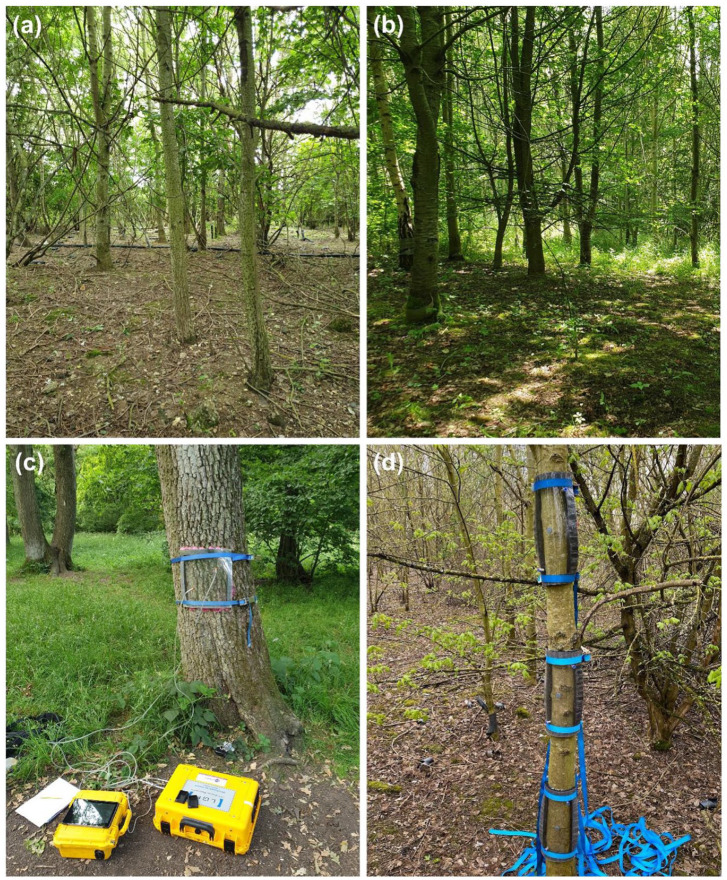
(a) The sampled landfill site, (b) the sampled non-landfill comparison site, (c) the gas sampling method which used a recirculating closed-loop system between gas flux chambers and a GHG analyser, and (d) gas flux chambers affixed to a tree.

GHG fluxes from trees were measured using a recirculating closed-loop system between gas flux chambers on the tree stem and a GHG analyser (ultraportable off-axis integrated cavity output spectroscopy analyser, Los Gatos Research) (See Figure 1 (c)) (Siegenthaler et al., 2016). We used semi-rigid flux chambers constructed from clear polycarbonate and closed-cell silicone foam strips with quick release connectors. They were secured to tree stems using ratchet straps and sealed with airtight putty. Flux measurements were taken from soil using rigid cylindrical chambers constructed from polyvinyl chloride. We inserted these chambers into the soil, and airtight putty was used where the outside of the chamber met the soil surface. A rigid polycarbonate lid with quick release connectors was secured to the top of the chambers, and this was connected to the GHG analyser.

The GHG analyser had a measurement range of 0.01–100 ppm ± 2 ppb for CH_4_, and a range of 1–20,000 ppm ± 300 ppb for CO_2_ measurements (Wilkinson et al., 2018). Changes in gas concentration were measured over 10-minute periods, during which time *ca.*600 measurements were taken. Typically, the first 100 seconds of each flux measurement time series was discarded to account for setup disturbances. A linear regression line was plotted for each data set, and the slope and *R*^2^ values were calculated. Gas fluxes were determined using the ideal gas equation and standardised for temperature and pressure. Positive flux values indicated emission and negative flux values showed the uptake of gas.

*R*^2^ values have been used in previous studies to identify the fluxes for further analysis, with data rejected if the *R*^2^ value was below 0.7 (Pangala et al., 2013; Welch et al., 2018). However, as many of the fluxes were close to the detection limits of the analyser, the *R*^2^ values were low despite patterns being observed in the time series graphs. In these cases, we used regression calculations in addition to the *R*^2^ values to determine their validity.

For upscaling measurements, the average tree surface area was calculated by considering the stem as a cylinder using an average tree stem diameter (of all measured trees) and a height of 3 m. The average surface area of one tree was multiplied by an estimated number of trees to determine the overall stem surface area on the site. An overall tree flux value was calculated from the product of the overall stem surface area and the average stem flux. The average soil surface flux and area of the site were multiplied to estimate the overall soil CH_4_ and CO_2_ fluxes. The magnitude of tree stem fluxes across the site was compared with soil emissions, and the percentage contribution of tree fluxes to the overall surface flux was calculated.

### Statistical tests

Graphs were produced using Origin (2020), and statistical tests were carried out in SPSS (24) and R (3.5.1). Repeated measures analysis of variance (ANOVA) tests were carried out when trees were measured multiple times over the field campaign and if the assumptions of normality (Shapiro–Wilk test) and equal variance (Levene’s test) were met. This was followed by multiple paired *t*-tests. A Friedman test was used for non-normal data, followed by a Wilcoxon Signed-rank test. Data that did not include multiple observations from the same individuals were analysed using a one-way ANOVA test (and a post hoc Tukey’s Honestly Significant Difference (HSD) test) for data with more than two groups, and *t*-tests where there were two groups. If single time point data were non-parametric, a Mann–Whitney *U*-test was used for two groups and a Kruskal–Wallis test for three or more groups. Full details of the statistical tests are displayed in Supplementary Table 1. Stepwise multiple regression analysis was carried out in SPSS (24) to evaluate the relationships between tree stem fluxes and measured environmental variables. In the landfill site data, air temperature was highly correlated with stem temperature and soil temperature (*R*^2^ > 0.9); therefore, these variables were excluded from the stepwise multiple regression analysis.

## Results and discussion

Average CH_4_ and CO_2_ tree stem emissions from all measurement heights at the former landfill site during the measurement period from August 2019 to February 2020 were 12.8 ± 34.5 µg m^–2^ h^–1^ and 53.3 ± 6.6 mg m^–2^ h^–1^, respectively. Mean soil CH_4_ and CO_2_ fluxes were 7.1 ± 20.3 µg m^–2^ h^–1^ and 256.0 ± 27.8 mg m^–2^ h^–1^, respectively.

### Temporal variations in gas fluxes

CH_4_ fluxes at the landfill site from tree stems at 90 cm varied significantly on a monthly basis (*p* < 0.05). CH_4_ flux values in September 2019 were significantly higher than those in October 2019 (*p* < 0.05), January 2020 (*p* < 0.01) and February 2020 (*p* < 0.05). On average, the magnitude and range of CH_4_ emission or uptake values in the months between August 2019 and December 2019 was larger than that of January and February 2020 (Table 1). This pattern was largely driven by one individual tree, hereafter referred to as Landfill High Flux Tree (LHFT), which emitted or took up a much larger amount of CH_4_ than other measured stems. The largest recorded emissions from the LHFT were at 30 cm in August 2019 (1966.6 µg m^–2^ h^–1^), before a switch to the largest uptake values occurred in November 2019 at 30 cm (−5997.3 µg m^–2^ h^–1^). The flux values subsequently rose again in December 2019, before fluxes of a considerably lower magnitude were recorded for the remainder of the measurement period (January and February 2020).

**Table 1.** CH_4_ and CO_2_ fluxes from tree stems at 90 cm and soil chambers on a closed landfill site between August 2019 and February 2020. Positive fluxes indicate emission and negative values show the uptake of gas.

	Month	CH_4_ (µg m^–2^ h^–1^)				CO_2_ (mg m^–2^ h^–1^)			
		Average	SE	Range	*n*	Average	SE	Range	*n*
Tree stem	Aug-19	63.8	64.5	1044.1	15	127.3	25.4	384.8	15
	Sep-19	99.4	71.2	1105.6	15	164.0	19.8	260.9	15
	Oct-19	26.9	28.2	444.7	15	10.7	8.2	111.2	15
	Nov-19	49.8	33.6	564.0	15	6.8	5.0	67.4	15
	Dec-19	92.5	82.3	1262.9	15	9.9	3.9	48.9	15
	Jan-20	0.5	1.4	25.1	15	3.0	3.1	41.8	15
	Feb-20	–2.6	5.1	94.4	15	5.3	3.0	39.3	15
Soil	Aug-19	–33.5	58.4	363.4	5	285.3	99.9	572.7	5
	Sep-19	37.2	22.8	124.2	5	524.2	59.9	365.1	5
	Oct-19	55.3	128.8	729.7	5	318.8	41.2	247.1	5
	Nov-19	–5.7	6.8	41.3	5	159.0	15.9	89.4	5
	Dec-19	–30.4	21.7	128.8	5	216.9	25.6	156.2	5
	Jan-20	–8.5	11.1	57.7	5	188.3	16.8	96.3	5
	Feb-20	35.3	39.0	238.8	5	99.4	18.3	99.6	5

SE: standard error.

Landfill surface CH_4_ fluxes are expected to be higher in winter because the rate of CH_4_ oxidation in cover soils is greater in summer (Börjesson and Svensson, 1997b; Chanton and Liptay, 2000; Rachor et al., 2013). However, in this study, the opposite was observed as average tree stem CH_4_ fluxes were 3.9 times higher in the summer months than winter months, with values of 72.7 ± 40.3 µg m^–2^ h^–1^ in the summer (August and September) and 18.5 ± 16.6 µg m^–2^ h^–1^ in the winter (December, January and February). This is similar to a natural ecosystem where biological processes are linked to changes in abiotic factors (e.g. light and temperature) (Pangala et al., 2015; Pitz et al., 2018; Terazawa et al., 2015; Wang et al., 2016). The seasonal change was most evident with tree stem fluxes, suggesting that CH_4_ tree stem emissions on landfill sites are not regulated by soil oxidation rates because this pathway bypasses methanotrophic bacteria and oxidation. CH_4_ fluxes from the soil were not significantly different between months during the observation period at the landfill site (*p* > 0.05).

CO_2_ fluxes from tree stems at the landfill site varied significantly between the months (*p* < 0.01). The average CO_2_ flux values in August and September 2019 were an order of magnitude higher than those in the months between October 2019 and February 2020, indicating a seasonal trend with greater fluxes in the summer months (Table 1). This concurs with results from studies in temperate upland and transitional ecosystems (Barba et al., 2019b; Pitz et al., 2018; Warner et al., 2017), where seasonal variations are likely driven by temperature-induced enzymatic changes in stem respiration (Damesin et al., 2002; Levy and Jarvis, 1998; Saveyn et al., 2008). Our results also agree with findings stating that temporal changes in CO_2_ fluxes are likely caused by variations in temperature, water availability, and tree physiology (Vargas and Barba, 2019).

Soil CO_2_ fluxes varied significantly between months (*p* < 0.05), with September and October fluxes considerably greater than those in late autumn and winter (November to February). This seasonal variation is likely due to increased soil respiration rates when temperatures were higher (Meyer et al., 2018). Larger CO_2_ fluxes in warmer months may also be partly due to higher soil CH_4_ oxidation rates resulting in increased CO_2_ production (Chanton and Liptay, 2000).

### Spatial variations in gas fluxes

#### Gas flux variations with measurement height

CH_4_ fluxes are expected to fall with increased tree height when tree stems are channelling an underground anaerobic source (Pangala et al., 2017; Rusch and Rennenberg, 1998; Terazawa et al., 2007). For example, average CH_4_ emissions from a forested floodplain in Japan were 176 and 97 mg m^–2^ h^–1^ from stem positions 15 and 70 cm above the ground, respectively (Terazawa et al., 2007). The average landfill tree stem CH_4_ flux (for all months in the measurement period) at 30 cm was –76.4 ± 141.8 µg m^–2^ h^–1^, whereas at 90 and 150 cm fluxes were 47.2 ± 19.0 µg m^–2^ h^–1^ and 21.9 ± 21.2 µg m^–2^ h^–1^, respectively (Supplementary Figure 2(a)). These contrasting measurements however were not statistically significant (*p* > 0.05) due to large variations in flux values at 30 cm.

Further analysis showed that there were spatial patterns in CH_4_ fluxes at different stem heights during some months. The average CH_4_ flux decreased with stem height in August 2019, whereas in November 2019 and February 2020, this pattern was not observed (Table 2). There was no significant difference between the CH_4_ fluxes from different stem heights in August, November or February (*p* > 0.05). During the intensive field measurements taken from 40 trees in February 2020, a pattern of decreasing fluxes with increased stem height (highest fluxes at 30 cm and lowest at 150 cm) was only recorded from six trees. Conversely, the opposite trend of increasing fluxes with stem height (highest emissions at 150 cm and lowest at 30 cm) was observed from 14 trees. The pattern of decreasing CH_4_ flux with stem height may be detectable in the summer rather than winter months as CH_4_ fluxes are generally larger in the warmer season (Pitz et al., 2018; Terazawa et al., 2015; Wang et al., 2016). The magnitude of this pattern may also be variable due to the lower permeability of the clay cap compared with the overlying cover soil which could cause anaerobic microsites to form due to hydrological changes above the cap (Environment Agency, 2014; Lewis et al., 2006). The observed occurrences of decreasing fluxes with stem height suggests that CH_4_ was moving up the tree stem via diffusion from an underground source during the summer months and may indicate that CH_4_ emitted by the trees originated from belowground.

**Table 2.** CH_4_ and CO_2_ fluxes at 30, 90 and 150 cm measurement heights during different months. Positive fluxes indicate emission and negative values show the uptake of gas.

Month	Measurement height (cm)	CH_4_ (µg m^–2^ h^–1^)			CO_2_ (mg m^–2^ h^–1^)				
		Average	SE	Range	*n*	Average	SE	Range	*n*
Aug-19	30	134.6	130.9	1977.3	15	154.3	27.7	418.5	15
	90	63.6	64.5	1044.1	15	127.3	25.4	384.8	15
	150	–6.7	13.6	242.6	15	192.5	28.9	407.2	15
Nov-19	30	–364.3	403.7	6474.8	15	12.4	6.6	80.9	15
	90	49.8	33.6	564.0	15	6.8	5.0	67.4	15
	150	70.8	61.5	1033.1	15	–12.1	7.4	92.6	15
Feb-20	30	0.4	3.9	67.5	15	8.9	4.4	64.4	15
	90	–2.6	5.1	94.4	15	5.3	3.0	39.3	15
	150	1.7	4.2	58.9	15	9.7	4.1	52.5	15

SE: standard error.

The same pattern of decreasing fluxes with increased stem height was also expected for CO_2_ if gas from the soil was transported via the transpiration stream of trees (Teskey et al., 2008). Average CO_2_ fluxes at each stem height (for all months in the measurement period) were positive. The largest mean emission of CO_2_ was measured at 150 cm (63.4 ± 17.0 mg m^–2^ h^–1^) and the lowest at 90 cm (46.7 ± 7.8 mg m^–2^ h^–1^); however, measured differences were not significant (*p* > 0.05; Supplementary Figure 2(b)). In November 2019, there was a statistically significant (*p* < 0.01) trend of decreasing fluxes with increased stem height (Table 2). The negative CO_2_ flux measured at 150 cm in November 2019 was likely due to the combined effect of a decreasing subsurface CO_2_ signature with height and a winter increase in stem photosynthesis (Saveyn et al., 2010).

In August 2019 and February 2020, the largest CO_2_ flux was at 150 cm, followed by 30 cm and the smallest was at 90 cm (Table 2). There were no significant differences between the CO_2_ fluxes from different stem heights in August, or February (*p* > 0.05). Larger CO_2_ emissions occur from the soil in the summer months when temperatures are higher, which is when the decreasing trend of CO_2_ fluxes with stem height would be expected to be the most prominent (Chanton and Liptay, 2000; Teskey et al., 2008). As this was not found, it is possible that other factors were altering this pattern. For example, tree stems may harbour methanotrophic bacteria which oxidise CH_4_ to produce CO_2_, this is then emitted from the tree stem at various heights (Covey and Megonigal, 2019; Jeffrey et al., 2021a, 2021b).

#### Spatial variations in gas fluxes at the landfill site

**Figure 2.**
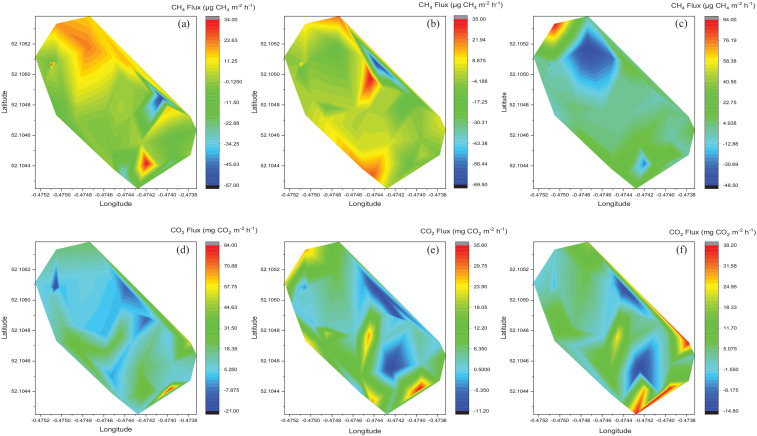
Contour plots showing spatial variation in CH_4_ and CO_2_ fluxes from tree stems at the landfill site during intensive sampling in February 2020. Positive fluxes indicate emission and negative values sho*w* the uptake of gas: (a) CH_4_ fluxes at 30 cm measurement height, (b) CH_4_ fluxes at 90 cm measurement height, (c) CH_4_ fluxes at 150 cm measurement height, (d) CO_2_ fluxes at 30 cm measurement height, (e) CO_2_ fluxes at 90 cm measurement height and (f) CO_2_ fluxes at 150 cm measurement height. Note different scale for each plot.

**Figure 3.**
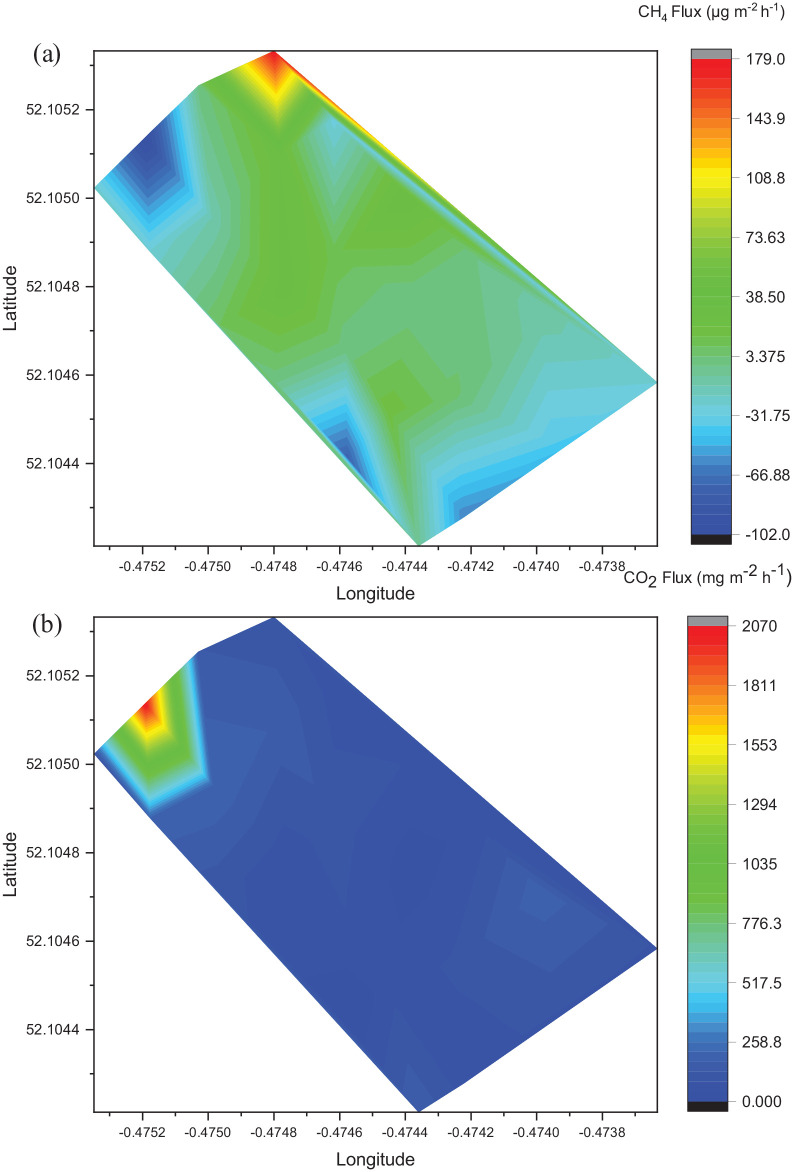
Contour plots showing spatial variation in CH_4_ and CO_2_ fluxes from soil at the landfill site during intensive sampling in February 2020. Positive fluxes indicate emission and negative values show the uptake of gas: (a) CH_4_ fluxes and (b) CO_2_ fluxes. Note different scale for each plot.

Spatial variations in stem fluxes (at each measurement height) and soil fluxes during the intensive February 2020 sampling visit are shown in Figure 3. The highest tree stem CH_4_ fluxes were predominantly in the north of the site with some isolated peaks in the south and south west at the 30 and 90 cm sampling heights (Figure 2(a) to (c)). The lowest tree stem CH_4_ fluxes at the 30 and 90 cm measurements heights were on the eastern edge of the site and the lowest fluxes at the 150 cm sampling height were in the north and north east with an isolated negative flux in the south. Unlike CH_4_, the largest tree stem CO_2_ fluxes were in the south of the site (Figure 2(d)–(f)). There was also a peak in CO_2_ fluxes from tree stems on the eastern edge of the site at the 150 cm measurement height. The lowest tree stem CO_2_ fluxes at 30 cm sampling height were in the north and east, whereas for the 90 and 150 cm measurement heights the lowest fluxes were in two areas in the south and east.

The highest CH_4_ fluxes from soils during the intensive sampling in February 2020 occurred in the north of the site (Figure 3(a)). The lowest soil CH_4_ fluxes were in the north west corner and towards the southern end of the site. On the contrary, the highest measured CO_2_ fluxes were in the north-west corner where CH_4_ fluxes had been the lowest (Figure 3(b)). The CO_2_ fluxes were otherwise relatively uniform across the site.

#### Gas flux variations between tree species

Stem CH_4_ fluxes were not significantly different between tree species (*Betula pendula, Fraxinus excelsior* and *Prunus avium*) at any measurement height (*p* > 0.05). The average CH_4_ flux from *Fraxinus excelsior* stems was generally an order of magnitude greater than that of the other species, but this was not significant due to the large range of flux values for this species (Table 3) and the inclusion of the LHFT.

**Table 3.** Summary of CH_4_ and CO_2_ fluxes at 30, 90 and 150 cm measurement heights from different tree species.

Species	Measurement height (cm)	CH_4_ (µg m^–2^ h^–1^)				CO_2_ (mg m^–2^ h^–1^)			
		Average	SE	Range	*n*	Average	SE	Range	*n*
*Betula pendula*	30	6.5	5.6	62.9	12	77.7	23.1	280.8	12
	90	15.9	7.2	189.1	28	57.5	18.2	453.0	28
	150	6.0	9.6	131.1	12	66.0	24.2	290.6	12
*Fraxinus excelsior*	30	–115.9	139.8	7963.9	30	49.7	17.9	489.3	30
	90	64.4	28.1	1406.3	70	38.0	8.2	252.9	70
	150	28.8	31.7	1048.3	30	54.8	21.0	546.5	30
*Prunus avium*	30	–13.6	10.4	34.1	3	70.1	64.2	194.8	3
	90	0.1	6.5	58.9	7	90.6	41.3	283.0	7
	150	16.9	7.1	24.1	3	138.3	120.9	377.4	3

Positive fluxes indicate emission and negative values show the uptake of gas.

SE: standard error.

Stem CO_2_ fluxes were not significantly different between any of the tree species at 30 or 150 cm (*p* > 0.05). CO_2_ emissions at 90 cm were significantly higher from *Betula pendula* than *Fraxinus excelsior* stems (*p* < 0.05). There was no significant difference in the stem CO_2_ fluxes between the other tree species at this measurement height.

#### Environmental controls on gas fluxes from tree stems

CH_4_ stem fluxes at 30 and 150 cm were not accounted for by any of the measured ancillary variables. At 90 cm, 28% of the variation in CH_4_ fluxes was explained by soil pH, tree DBH and soil bulk density (Supplementary Table 3). Stem CO_2_ fluxes at 30 cm were best explained by air temperature, which accounted for 51% of the variation (Supplementary Table 3). At 90 cm from ground level, 57% of the variation in CO_2_ fluxes was explained by air temperature and air pressure. CO_2_ fluxes at 150 cm were best accounted for by air temperature (66%) (Supplementary Table 3). The averages and ranges of the measured environmental variables for the landfill and non-landfill site are in Supplementary Table 2. Full results of the stepwise regression analysis are in Supplementary Table 3.

#### Variations in gas fluxes from landfill

Average fluxes from trees and soils indicated that the landfill site was a net source of CH_4_ and CO_2_ during the measurement period from August 2019 to February 2020. Landfill soil CH_4_ measurements ranged from –230 µg m^–2^ h^–1^ to 557 µg m^–2^ h^–1^, which agreed with previous landfill CH_4_ surface fluxes in terms of variability and magnitude (−0.02 to over 4000 g m^–2^ d^–1^) (Boeckx et al., 1996; Bogner et al., 1997; Börjesson and Svensson, 1997a; Rachor et al., 2013; Scharff and Jacobs, 2006). Some sampled trees and soil locations emitted substantially more CH_4_ than others (Figure 3). This variation may be explained by localised sources such as poor drainage in some areas, a leak in the landfill cap or gas extraction pipe, or unrecorded variations in management practices at these times (e.g. extraction system malfunctions or maintenance).

A comparison of landfill data from August 2019 and February 2020 with non-landfill data from February and August 2020 showed that CH_4_ fluxes from tree stems were marginally different between the sites (*p* = 0.065). The average stem CH_4_ fluxes from the landfill and non-landfill sites during these months were 31.8 ± 24.4 µg m^–2^ h^–1^ and −0.3 ± 0.2 µg m^–2^ h^–1^, respectively. The large difference in average values is due to summer season landfill site tree fluxes which were orders of magnitude larger than those from the non-landfill comparison site. Further analysis due to the bimodal distribution of the data showed that there was no significant difference in stem CH_4_ emissions at any measurement height in February or August between the landfill and non-landfill site (*p* > 0.05). There was also no significant difference between the soil CH_4_ emissions from the landfill and non-landfill site in either February or August (*p* > 0.05).

The magnitude and range of stem CO_2_ flux values for the landfill and non-landfill sites were similar (Table 4). In the summer season, there was no significant difference in stem CO_2_ fluxes between the two sites at 30 and 90 cm (*p* > 0.05). At 150 cm, there was a significant difference between the CO_2_ emissions in August, with the landfill site trees emitting more CO_2_ on average (*p* < 0.05) (Table 4). In the winter season, there was no significant difference in stem CO_2_ fluxes between the landfill and non-landfill site at any of the measurement heights (*p* > 0.05). There was also no significant difference in soil CO_2_ emissions in August between the two sites (*p* > 0.05). In February, soil CO_2_ emissions from the non-landfill site were significantly larger than those from the closed landfill (*p* < 0.01). The average stem CH_4_ flux for the landfill site is of the same order of magnitude as the lowest end of the range of fluxes from trees on temperate wetland sites (Gauci et al., 2010; Pangala et al., 2013) and one study from a temperate upland area (Pitz et al., 2018). Measurements were however considerably more varied leading to a larger range in flux values compared to natural ecosystems; this is likely due to the trees being planted on landfill, where there is a high natural variability of fluxes (Bogner et al., 1997; Pangala et al., 2013, 2015). The observed variability within the landfill system makes identifying the source of the emitted CH_4_ challenging. Future investigations could use isotopic approaches to determine the origin (heartwood rot or belowground source) of CH_4_ emitted by tree stems on landfill sites (Barba et al., 2019a).

**Table 4.** Summary of CH_4_ and CO_2_ fluxes in a closed landfill (August 2019 and February 2020) and a non-landfill area (February and August 2020).

	Measurement type	CH_4_ (µg m^–2^ h^–1^)				CO_2_ (mg m^–2^ h^–1^)			
		Average	SE	Range	*n*	Average	SE	Range	*n*
Closed landfill site August 2019	Tree stem all heights	63.8	48.5	2154.1	45	158.0	16.0	488.2	45
	Tree stem 30 cm	134.6	130.9	1977.3	15	154.3	27.7	418.5	15
	Tree stem 90 cm	63.6	64.5	1044.1	15	127.3	25.4	384.8	15
	Tree stem 150 cm	–6.7	13.6	242.6	15	192.5	28.9	407.2	15
	Soil	–33.5	58.4	363.4	5	285.3	99.9	572.7	5
Non-landfill site August 2020	Tree stem all heights	–0.3	0.2	8.8	45	140.3	18.5	602.2	45
	Tree stem 30 cm	–0.8	0.5	8.5	15	158.2	41.5	602.2	15
	Tree stem 90 cm	0.0	0.1	0.8	15	156.2	27.8	323.7	15
	Tree stem 150 cm	–0.2	0.1	1.8	15	106.4	24.6	369.2	15
	Soil	–43.7	3.7	20.1	5	403.0	36.9	200.9	5
Closed landfill site February 2020	Tree stem all heights	–0.2	2.5	102.5	45	8.0	2.2	67.8	45
	Tree stem 30 cm	0.4	3.9	67.5	15	8.9	4.4	64.4	15
	Tree stem 90 cm	–2.6	5.1	94.4	15	5.3	3.0	39.3	15
	Tree stem 150 cm	1.7	4.2	58.9	15	9.7	4.1	52.5	15
	Soil	35.3	39.0	238.8	5	99.4	18.3	99.6	5
Non-landfill site February 2020	Tree stem all heights	–0.2	0.2	8.1	45	4.9	1.5	58.7	45
	Tree stem 30 cm	–0.3	0.3	4.4	15	10.2	3.1	51.8	15
	Tree stem 90 cm	–0.1	0.4	5.8	15	2.1	2.0	26.3	15
	Tree stem 150 cm	–0.2	0.4	7.5	15	2.4	2.5	37.4	15
	Soil	–9.9	2.8	16.8	5	183.4	13.1	73.4	5

Positive fluxes indicate emission and negative values sho*w* the uptake of gas.

SE: standard error.

Variation in stem fluxes on the landfill site is largely explained by individual trees. For example, the flux from the LHFT was almost 25 times higher than the average flux in August 2019. The magnitude of this flux is comparable with the largest CH_4_ emission recorded in a temperate floodplain forest by Terazawa et al. (2015), who also reported high variability in flux measurements. However, the soil effluxes from locations near LHFT did not display similar patterns to the tree stem CH_4_ fluxes, indicating that high rates of soil CH_4_ oxidation prevented large soil surface emissions. It is possible that this tree stem fluctuated between being a source or sink of CH_4_ depending on nearby landfill conditions. For example, the response of methanotrophs to a change in CH_4_ source strength would result in a temporary increase in CH_4_ oxidation rates and would explain the sudden switch from emission to uptake by LHFT (Bender and Conrad, 1995; Cai et al., 2016). This influx of CH_4_ in the vicinity of LHFT could be caused by a leak in the landfill cap or disruption to the gas extraction system.

The upscaled landfill soil surface flux from our experimental site was 759 g CH_4_ yr^–1^ and the stem CH_4_ flux was 493 g CH_4_ yr^–1^; the soil surface flux was comparable to the lower end of the range of fluxes from a landfill site with active gas extraction (−0.02 to 70.1 g m^–2^ d^–1^) (Börjesson and Svensson, 1997a). In this investigation, tree stem emissions accounted for 39% and 7% of the estimated total (soil and tree stem) landfill surface emissions of CH_4_ and CO_2_, respectively. This is greater than the contribution of tree emissions to the total ecosystem CH_4_ flux in a temperate forested wetland (up to 27%), but substantially lower than the contribution in a tropical forested wetland (up to 87%) (Pangala et al., 2013, 2015). Carbon released through tree stems to the atmosphere may offset a proportion of the carbon sequestration and stem CH_4_ fluxes need to be factored into carbon assessments for managed environments. This investigation identifies the need to include tree stem emissions when quantifying the total surface fluxes from landfill environments; without the inclusion of tree emissions, flux values are unlikely to be accurate.

## Conclusion

This study has revealed that tree stem fluxes accounted for 39% and 7% of the total landfill surface CH_4_ and CO_2_ emissions, respectively. Fluxes from the landfill site were highly variable among different trees, areas of the site and between different site visits, which concurs with the high spatial and temporal variability previously recorded from landfill surfaces. There was evidence in some months to support that trees were facilitating the transport of CH_4_ from the soil to the atmosphere. In addition, localised or intermittent CH_4_ emissions indicated that the largest emissions may be explained by landfill conditions (such as leaks in the cap, anaerobic microsites in the soil zone above the cap or changes to landfill management procedures), rather than alternative environmental controls including temperature and soil moisture near the surface. Our findings demonstrate that some tree species planted on landfill have the capacity to emit more CH_4_ than they would otherwise if planted in a more natural setting. Results also indicate that measuring soil fluxes alone from a forested landfill site would result in an underestimation of the total surface flux. There is a need to understand the mechanisms responsible for stem emissions from landfill trees. This would both constrain observed variability and enable mitigation of any such legacy emissions from former landfill sites. These results suggest that trees planted on former landfill sites are altering terrestrial GHG fluxes.

## Supplemental Material

sj-docx-1-wmr-10.1177_0734242X221086955 – Supplemental material for Methane emissions from trees planted on a closed landfill site Click here for additional data file. Supplemental material, sj-docx-1-wmr-10.1177_0734242X221086955 for Methane emissions from trees planted on a closed landfill site by Alice Fraser-McDonald, Carl Boardman, Toni Gladding, Stephen Burnley and Vincent Gauci in Waste Management & Research
