# Supplementary material for: Methane emissions from trees planted on a closed landfill site
Source: Waste Manag Res. 2022 Apr 5;40(11):1618–28. doi: 10.1177/0734242X221086955 (PMC9580030; doi:10.1177/0734242X221086955)
Supplement: sj-docx-1-wmr-10.1177_0734242X221086955 – Supplemental material for Methane emissions from trees planted on a closed landfill site [file sj-docx-1-wmr-10.1177_0734242X221086955.docx]

**Supplementary Information**

**Supplementary Methods**

*Spatial analysis of gas fluxes at the landfill site*

Spatially intensive fieldwork in which 40 trees and 24 soil locations were sampled was carried out at the landfill site in February 2020. Measurements were taken between 08:00 and 16:00 within the space of 10 days. Tree stems were sampled at 30 cm, 90 cm and 150 cm measurements heights. Origin (2020) software was used to create contour plots to display the magnitude of fluxes across the site. This software uses a four-step process to create these plots. Firstly, triangulation is performed which involves connecting the data points in the XY plane to create Delaunay triangles. Linear interpolation is then carried out and the intersection points of contour lines and the sides of the triangles are found; these are marked as characteristic points. Next, the contour lines are drawn by tracing the characteristic points. Finally, the characteristic points are connected and smoothing takes place. Contour plots were created for CH_4_ and CO_2_ measurements at each stem height and for soil flux locations.

**Supplementary Results**

**Supplementary Table 1** Details of the statistical tests used for each data set including normality of data, the tests used, and the resultant test and p values.

| **Data** | **Parametric/Non-parametric** | **Statistical Test** | **Test Statistic** | **P value** |
| --- | --- | --- | --- | --- |
| Monthly comparison of CH_4_ stem fluxes from the landfill site | Non-parametric | Friedman test (post-hoc: Wilcoxon signed-rank test) | 13.82 | p < 0.05 |
| Monthly comparison of CH_4_ soil fluxes from the landfill site | Non-parametric | Friedman test (post-hoc: Wilcoxon signed-rank test) | 7.08 | p < 0.01 |
| Monthly comparison of CO_2_ stem fluxes from the landfill site | Non-parametric | Friedman test | 58.45 | p > 0.05 |
| Monthly comparison of CO_2_ soil fluxes from the landfill site | Parametric | Repeated measures ANOVA (post-hoc: paired t-test) | 7.31 | p < 0.05 |
| Comparison of CH_4_ fluxes at different stem heights from the landfill site (all months) | Non-parametric | Kruskal-Wallis test | 1.22 | p > 0.05 |
| Comparison of CO_2_ fluxes at different stem heights from the landfill site (all months) | Non-parametric | Kruskal-Wallis test | 0.69 | p > 0.05 |
| Comparison of CH_4_ fluxes at different stem heights from the landfill site (August 2019) | Non-parametric | Kruskal-Wallis test | 0.07 | p > 0.05 |
| Comparison of CO_2_ fluxes at different stem heights from the landfill site (August 2019) | Parametric  (after sqrt transform) | One-way ANOVA | 2.18 | p > 0.05 |
| Comparison of CH_4_ fluxes at different stem heights from the landfill site (November 2019) | Non-parametric | Kruskal-Wallis test | 0.93 | p > 0.05 |
| Comparison of CO_2_ fluxes at different stem heights from the landfill site (November 2019) | Parametric  (after sqrt transform) | One-way ANOVA (post-hoc: Tukey’s test) | 5.23 | p < 0.01 |
| Comparison of CH_4_ fluxes at different stem heights from the landfill site (February 2020) | Non-parametric | Kruskal-Wallis test | 0.62 | p > 0.05 |
| Comparison of CO_2_ fluxes at different stem heights from the landfill site (February 2020) | Parametric  (after sqrt transform) | One-way ANOVA | 0.23 | p > 0.05 |
| Comparison of CH_4_ stem fluxes between tree species at the landfill site | Non-parametric | Kruskal-Wallis test | 0.39 | p > 0.05 |
| Comparison of CO_2_ stem fluxes between tree species at the landfill site | Non-parametric | Kruskal-Wallis test (post-hoc: Dunn’s test) | 13.97 | p < 0.01 |
| Comparison of CH_4_ stem fluxes between the landfill and non-landfill sites | Non-parametric | Mann-Whitney U test | 3410.00 | p = 0.065 |
| Comparison of CO_2_ stem fluxes between the landfill and non-landfill sites | Non-parametric | Mann-Whitney U test | 3716.00 | p > 0.05 |
| Comparison of CH_4_ soil fluxes between the landfill and non-landfill sites | Parametric | One-way ANOVA | 0.61 | p > 0.05 |
| Comparison of CO_2_ soil fluxes between the landfill and non-landfill sites | Parametric  (after sqrt transform) | One-way ANOVA | 3.44 | p > 0.05 |
| Comparison of CH_4_ stem fluxes between the landfill and non-landfill sites (August 2019 only) | Non-parametric | Mann-Whitney U test | 849.00 | p > 0.05 |
| Comparison of CO_2_ stem fluxes between the landfill and non-landfill sites (August 2019 only) | Non-parametric | Mann-Whitney U test | 933.00 | p > 0.05 |
| Comparison of CH_4_ soil fluxes between the landfill and non-landfill sites (August 2019 only) | Parametric | One-way ANOVA | 0.03 | p > 0.05 |
| Comparison of CO_2_ soil fluxes between the landfill and non-landfill sites (August 2019 only) | Parametric | One-way ANOVA | 1.22 | p > 0.05 |
| Comparison of CH_4_ stem fluxes between the landfill and non-landfill sites (February 2020 only) | Non-parametric | Mann-Whitney U test | 846.00 | p > 0.05 |
| Comparison of CO_2_ stem fluxes between the landfill and non-landfill sites (February 2020 only) | Parametric  (after sqrt transform) | One-way ANOVA | 0.76 | p > 0.05 |
| Comparison of CH_4_ soil fluxes between the landfill and non-landfill sites (February 2020 only) | Parametric | One-way ANOVA | 1.33 | p > 0.05 |
| Comparison of CO_2_ soil fluxes between the landfill and non-landfill sites (February 2020 only) | Parametric | One-way ANOVA | 13.94 | p < 0.01 |

**Supplementary Figure 2 (a)** Bar chart showing mean CH_4_ fluxes at each measured stem height; **(b)** Bar chart showing mean CO_2_ fluxes at each measured stem height. Error bars show ±1 SE.

**Supplementary Table 2** Average, minimum, maximum and ranges of measured environmental variables at the landfill and non-landfill sites.

| **Field site** | **Environmental variable** | **Average** | **Minimum** | **Maximum** | **Range** |
| --- | --- | --- | --- | --- | --- |
| **Landfill** | Air temperature (°C) | 12.1 | 0.9 | 29.5 | 28.6 |
|  | Air pressure (hPa) | 1004.1 | 991.3 | 1019.0 | 27.7 |
|  | Stem temperature (°C) | 11.8 | 1.2 | 25.8 | 24.6 |
|  | Soil temperature (°C) | 10.3 | 4.2 | 20.5 | 16.3 |
|  | Soil moisture (%) | 25.1 | 5.3 | 44.7 | 39.4 |
|  | Stem DBH (cm) | 17.1 | 10.2 | 39.7 | 29.5 |
|  | Soil pH | 7.0 | 6.7 | 7.3 | 0.6 |
|  | Soil bulk density (g cm^-3^) | 1.1 | 0.9 | 1.3 | 0.4 |
| **Non-landfill** | Air temperature (°C) | 14.0 | 4.8 | 23.7 | 18.9 |
|  | Air pressure (hPa) | 1008.6 | 999.2 | 1019.8 | 20.6 |
|  | Stem temperature (°C) | 12.8 | 3.1 | 20.7 | 17.6 |
|  | Soil temperature (°C) | 10.5 | 4.0 | 17.0 | 13.0 |
|  | Soil moisture (%) | 32.0 | 10.2 | 48.6 | 38.4 |
|  | Stem DBH (cm) | 16.1 | 12.7 | 21.4 | 8.7 |
|  | Soil pH | 7.1 | 6.9 | 7.3 | 0.4 |
|  | Soil bulk density (g cm^-3^) | 0.9 | 0.7 | 1.0 | 0.3 |

**Supplementary Table 3** Results of stepwise regression analysis showing the relationships between CH_4_ and CO_2_ fluxes, and environmental variables at the landfill site (at different stem heights).

|  | **Height** | **Adjusted R^2^** | **Explanatory variables** | **p value** | **Standardised β coefficient** |
| --- | --- | --- | --- | --- | --- |
| **CH_4_ stepwise regression model** | 30 cm | - | - | - | - |
|  | 90cm | 0.280 | Soil pH | p < 0.01 | 0.530 |
|  |  |  | Tree DBH | p < 0.01 | -0.368 |
|  |  |  | Soil bulk density | p < 0.01 | -0.276 |
|  | 150 cm | - | - | - | - |
| **CO_2_ stepwise regression model** | 30 cm | 0.507 | Air temperature | p < 0.01 | 0.720 |
|  | 90 cm | 0.571 | Air temperature | p < 0.01 | 0.741 |
|  |  |  | Air pressure | p < 0.01 | -0.279 |
|  | 150 cm | 0.663 | Air temperature | p < 0.01 | 0.819 |
